# Dog Tales: Mindful Dog Interactions Evoke Similar Experiences to Dog Assisted Mindfulness Meditations

**DOI:** 10.3390/ani11072104

**Published:** 2021-07-15

**Authors:** Jessica Lee Oliva, Tim Robert Green

**Affiliations:** 1 School of Psychological Sciences, Faculty of Medicine, Nursing and Health Sciences, Monash University, Melbourne 3800, Australia; tim-green@bigpond.com; 2 College of Healthcare Science, James Cook University, Townsville 4811, Australia

**Keywords:** dog, mindfulness, meditation, COVID-19, AAI, interactions, loneliness, owner, pet

## Abstract

**Simple Summary:**

As humans went into lockdown in response to the global COVID-19 pandemic, so did their pets. The resulting loss of human freedoms can be an important reflection point for standard pet keeping practices. This study evaluated the feasibility and effectiveness of two six-week interventions that owners can complete with their pet dogs from the safety and comfort of their homes, designed to enhance the wellbeing of both parties. One was a dog-assisted mindfulness intervention, whereby participants would sit quietly with their dog and listen to a mindfulness recording, using a feature of their dog as their object of focus, e.g., their dog’s fur. The other was a dog interactions intervention, whereby participants spent at least 7 min of undivided attention interacting with their dog in different ways each week. Common experiences were reported across the two interventions including: enhanced owner–dog connection, and feelings of relaxation, happiness and engagement both during and after participating in the weekly activities. Additionally, ‘dog happiness’ was commonly reported in the dog interactions group. Using our own experiences of being “locked down” as a reference point, this study offers two novel ways in which owners can attempt to enrich the lives of their dogs at home.

**Abstract:**

Stay-at-home regulations in response to COVID-19 have put humans at increased risk of loneliness. Some studies support dog ownership as a protection against loneliness, while other studies have suggested the lockdowns can be used to reflect upon the similar restrictions owners impose on their pets on a daily basis. This study evaluated two novel ways to enrich the lives of pet dogs in the home, while also providing benefits to owners. It was hypothesized that a six-week Dog Assisted Mindfulness (DAM) intervention and a Dog Interactions (DI) intervention would positively impact owner-rated loneliness, mindfulness, and owner–dog emotional attachment, compared to a control group. Seventy-three participants were randomly assigned to each group. Mixed methods ANOVAs found no significant main effects of group, nor any group × time interaction effects. Qualitative analyses revealed common experiences among participants in the two active interventions, including enhanced owner–dog connection, and feelings of relaxation, happiness and engagement both during and after participating in the weekly activities. There was also an added benefit of ‘dog happiness’ in the interactions group. Future studies should investigate this in a more objective manner and in the meantime, regular owner–dog interactions should be encouraged, especially during times of extended lockdown.

## 1. Introduction

Following the outbreak of COVID-19, government enforced lockdowns have regulated social behaviours in an attempt to keep humans physically safe from harm. However, home confinement has been psychologically challenging for many people, and has resulted in freedom protests around the globe (for example, [1]). Hockenhull and Furtado [2] have suggested that this is an opportune time to reflect on the confinements and lack of choice we offer to domesticated animals, and whether such self-reflections could lead us to improve their welfare. Using horses as an example, they draw parallels between the “gold standard” industry conditions in which they are kept in “luxurious stables”, but robbed of the freedom to move as they please, eat when they please, socialise as they please, etc. We can extend this analogy to domesticated dogs living in our home. They too are often considered to live in luxurious conditions with all the home comforts we can offer them—a warm bed, warm clothes, even an assortment of toys, but they are usually not free to come and go as they please, eat when they please or socialise when they please. Humans were able to get sense of how this may feel during the COVID-19 related lockdowns. In many countries including Australia, one hour of daily exercise has been permitted during lockdown periods. For many this became the highlight of the day, and allowed owners to appreciate the excitement they see dogs express when asked if they would like to go on their daily walk.

Dogs typically show similar displays of excitement when their owners return home to them after a period of absence, and their clear lack of resentment for this has long led humans to believe in the status quo of pet-keeping. Indeed, such displays of excitement and affection lead many owners to believe their pets love them, and it has been shown that dogs are capable of forming attachment bonds similar to human caregiver-infant bonds [3,4,5,6]. However, it is important to consider if it is love based on reciprocity, freedom, and choice. Like Hockenhull and Furtado [2], we see the need to consider what more we can do to enrich the lives of domesticated species, using our own experiences of the COVID-19 lockdowns as a reflection point. Owner-pet dog interactions where “the human’s attention was completely focused on the dog” have been shown to increase the release of molecules associated with relaxation and reward in both parties [7] (p. 297), and hence, increasing such interactions may be one way in which this can be achieved. Similar physiological findings have also been observed in trainee assistance dogs following human interactions [8]. Human interaction with dogs has also been applied in shelter situations and has been shown to reduce stress related behaviours as well as plasma cortisol, which is believed to reflect stress related activation of the Hypothalamic Pituitary Adrenal (HPA) axis [9,10].

From the human perspective, a ‘must love dogs’ social initiative has been suggested as a potential solution to loneliness experienced by senior Australian citizens living alone prior to the pandemic [11], and dog ownership was recently shown to be protective against loneliness for people living alone during COVID-19-related lockdowns in Australia [12]. Contrary to expectations, there was no relationship between frequency of dog interactions and loneliness, nor between frequency of dog interactions and mindfulness, however, there was a negative relationship between mindfulness and loneliness, as has been previously reported [13,14]. Mindfulness practice originated from ancient Buddhist teachings which promotes being aware of the present moment and accepting what is experienced from a non-judgemental perspective [15]. The general guiding principle of mindfulness involves the focus on the now, rather than the tendency to ruminate about past events or worry about future events [15]. Oliva and Johnston’s [12] null findings relating to dog interactions were unexpected because it was originally proposed by the authors that mindfulness would mediate the relationship between frequency of dog interactions and loneliness in the following way. First, the individual interacts with their dog, due to the nature of these interactions the mind becomes one pointed and attentive towards its object of focus, the dog, thereby increasing the individual’s state of mindfulness, which in turn, reduces feelings of loneliness. However, Oliva and Johnston relied on cross-sectional data and the reliability of the scale used to measure frequency of dog interactions did not reach an acceptable level, which may reflect issues with its sensitivity.

In a randomised controlled trial, Shearer et al. [16] assigned undergraduate psychology students into three groups, a mindfulness practice group, a dog interaction group without mindfulness practice, and a no intervention control group. At the end of their four-week study, the researchers observed significant improvements in anxiety and mood states for both the mindfulness, and just dog interaction group, but not for the control group. The findings from this study suggest both mindfulness practices and dog interactions act as effective techniques for the self-management of wellbeing, which may also act as a protective barrier against loneliness. Henry and Crowley [17] found that including dogs within mindfulness practice did not result in any additional benefits when compared to a mindfulness practice alone in terms of reduction in symptoms of psychological distress, or the acquisition of mindfulness skills, however, participants did report higher ratings of the training and endorsed future participation more highly in the dog assisted mindfulness group. Considering how challenging mindfulness practices can be to learn and maintain (for example, [18,19]), the presence of a dog may help with participant adherence to a mindfulness program and/or increase motivation and engagement.

The aim of this study then was to determine whether engaging in a Dog Assisted Mindfulness (DAM) six-week intervention would be associated with better mental health outcomes (i.e., reduced loneliness, increased mindfulness, and increased emotional attachment to pet) as compared to a Dog Interaction (DI) and control group. It was hypothesized that both DAM and DI groups would experience lower levels of loneliness, and increased mindfulness and emotional attachment levels, as compared to the control group upon completion of the six-week study. From a qualitative perspective, we also explored how the experience of a DAM intervention compares with a DI intervention over a six-week study period.

## 2. Method

### 2.1. Participants

Participants were invited to take part if they were adults (18+), Australian residents/citizens and owned a dog. Promotion of the study occurred via social media and personal networks, with no payment or incentive offered for participation. Seventy-three participants enrolled in the study and were randomly assigned based on order of enrolment, to either one of three conditions: (i) a DAM group (*n =* 24), which involved participating in an approximate 7 min audio weekly guided mindfulness practices (ii) a DI group (*n =* 25), which involved interacting with their pet dog in a particular way for approximately 7 min or (iii) a control group (*n =* 24), which involved no prescribed weekly activity.

Two participants withdrew part way through the study, one from the DAM group because their dog passed away, and one from the DI group because they felt their dog was too young to participate in the activities, leaving a total sample of 71. Of these two from the DAM group, one from the DI group, and one from the control group did not return their baseline survey. Demographic information relating to the remaining 67 participants can be seen in Table 1 below. The state of Victoria is separated into Metropolitan and Regional because at the study’s commencement Metropolitan Victoria was under stage 4 lockdown restrictions, and Regional Victoria was under stage 3 lockdown restrictions. In both cases, there were only four valid reasons to be out of one’s house including: (i) shopping for food, (ii) accessing medical services or providing care, (iii) exercise, and (iv) going to work as an “essential worker”. Additionally, stage 4 included an 8 p.m.–5 a.m. curfew, a 1 h exercise limit, restrictions on movement beyond a 5 km radius of one’s home without a permit, and mandatory mask wearing anywhere outside of the home. Participant ages ranged from 26–79, with the mean age 43.4 (±11.8), which was similar across the three groups (DAM = 43.4 (±13.4), DI = 42.4 (±11.1), control = 44.4 (±11.24)).

### 2.2. Measures

Participants completed a baseline survey including demographic information such as participants’ living arrangements and state, age, gender, education, and previous mindfulness training experience. Participants were then asked to answer questions relating to four measures shown below.

The Perceived Emotional Closeness subscale of the Monash Dog Owner Relationship Scale (MDORS; [20]) consists of 10-items used to measure how dog owners feel about their pet dogs (e.g., ‘I wish my dog and I never had to be apart’). Statements are answered using a five-point Likert-type scale ranging from ‘Strongly Agree’ to ‘Strongly Disagree’. The five-point Likert type scale is different for two of the 10 questions, including ‘How often do you tell your dog things you don’t tell anyone else?’, which is rated from ‘Once a day’, to ‘Never’, and ‘How traumatic do you think it will be for you when your dog dies?’ rated from ‘Very traumatic’ to ‘Very untraumatic’. Scores are reversed, summed, and then averaged and so range from 1–5 with higher scores indicating higher emotional closeness. This subscale has previously demonstrated good internal consistency (α = 0.84) (e.g., [20]). Reliability in the current study was found to be acceptable at baseline (α = 0.77) and at post-intervention (α = 0.76).

The Freiburg Mindfulness Inventory (FMI; [21]) short 14 item version was used to measure participants mindfulness during a specific time frame (e.g., I am open to the experience of the present moment). The FMI uses a four-point Likert-type scale ‘rarely’, ‘occasionally’, ‘fairly often’ and ‘almost always’ with higher scores representing higher mindfulness. In this study, we asked participants to consider their experience with mindfulness during the ‘last two weeks’. Scores are summed and range between 14 and 56 with higher scores indicating higher mindfulness. Previous research using this scale has demonstrated good reliability (α = 0.86) (e.g., [21]). Reliability in the current study was also found to be good at baseline (α = 0.88) and at post-intervention (α = 0.88).

The 3-item University of California, Los Angeles Loneliness scale (UCLA-LS; [22]) is a short version of the longer questionnaire developed by Russell et al. [23] that measures perceived loneliness (i.e., I’ve felt isolated from others). The scale was rated on a four-point Likert-type scale with available responses, ‘never’, ‘rarely’, ‘sometimes’, ‘often’. We asked participants to consider their responses based on ‘the last two weeks’ which aligned with the mindfulness scale above. Scores are summed and range between 3 and 12 with higher scores indicating higher loneliness. Previous research using this scale has demonstrated good reliability (α = 0.72) (e.g., [22]). Reliability in the current study was also found to be good at baseline (α = 0.83) and at post-intervention (α = 0.89).

A “direct”, 1-item measure of loneliness was also used, in line with recommendations from the Office of National Statistics [24], and recommendations for measuring loneliness in a pet owner sample [25,26]. This measure asked participants to rate how often they had felt lonely in the past two weeks, and was again rated on the same 4-point Likert scale as the UCLA-LS.

We also asked participants from the DAM group and DI group how often they completed the assigned task throughout the week, and used Qualtrics to capture their freehand responses about their experiences, including how they felt during the task, or how they felt any time before or after it.

### 2.3. Materials

The DAM script was designed by the research team and informed by Williams et al. [27] who provides helpful guidance with structuring mindfulness script development. The DAM scripts were based on guided imagery which involves activating most of the senses (i.e., sight, touch, hearing) as instructed by Utay and Miller [28]. When these senses are activated during mindfulness practice, participants should experience higher levels of engagement [29]. Soft meditative music played in the background and the sound of bell/chime signalled the beginning and the end of each practice.

### 2.4. Procedure

Before commencement of the study ethical approval was obtained from the Monash University Human Research Ethics Committee. Interested individuals who came across the study on social media or via email contacted the research team and were provided with an explanatory statement and consent form. Once signed consent forms were returned, participants received a ‘Welcome email’ confirming their enrolment and study ID number. Participants were emailed every Friday afternoon with instructions for the week’s task depending on which group they were assigned to (including the audio recording if they were in the DAM group). The control group was also emailed weekly with a message thanking them for their participation and telling them there was nothing in particular they needed to do this week and that the research team would check back in with them in a week’s time. For all groups, the emails also contained links to the online questionnaires which participants were required to complete at week 1 and 7 and were accessed by clicking on a secure link to a Qualtrics platform hosting the study. The DAM group and DI group also received additional weekly questionnaires to complete about their experiences with the prescribed task. Participants in the DI group participated in simple dog interactions that are generally well known activities such as playing hide and seek. These dog interactions were designed by the research team based on personal experiences. Participants in the DAM group were instructed to create a space where they could sit uninterrupted and to turn off their phone. In weeks 1–5, before beginning the practice they were told to invite their dog to sit either next to them on a blanket or bed, or on their lap. In week 6 they were encouraged to complete the task in the absence of their dog. The DI group were told to focus on their dog and turn off their phone across all 6 weeks. An outline of the weekly activities is summarised in Table 2.

Participants in the DI group were told they could complete the task as many time as they pleased during the week. Similarly, participants in the DAM group were also told them same, and that they could complete it with or without the recording. Furthermore, they were told that, as the practices encourage tapping into one’s senses to become more aware of one’s dog, that they could do it anytime, even when their dog is not with them.

### 2.5. Design and Analysis

The research utilised a mixed method randomised controlled trial design. The dependent variables were participants’ perceived emotional closeness to their dogs, mindfulness, and perceived loneliness. Participant group allocation was decided according to a predetermined, pseudo randomised and counterbalanced chart. Neither the participants nor the researchers had a choice in group allocation. However, at the cessation of the study, all participants gained access to the tasks of the active intervention groups to complete in their own time if they wished. To analyse the effectiveness of the interventions on the above dependant variables, group differences across time were analysed using a series of mixed methods ANOVAs after data from the three time points were exported directly from Qualtrics into SPSS v 26 and combined. Participant free hand responses regarding their experiences of the weekly activities were analysed using a thematic content analysis. The two authors of this paper (JLO and TRG) independently coded these responses after each week and then met and compared coding and theme names, and resolved any discrepancies through discussion. Participants could endorse more than one theme with separate segments of their response, but could not endorse the same theme twice in the same week. Only themes endorsed by ≥5% of the sample who provided responses in any given week were retained. The sample size for each question included participants who provided responses that loaded on these themes, as well as responses that did not load on these main themes but that still provided an appropriate response to the question. Participants who did not provide a response or who provided a response that did not adequately address the question were not included.

## 3. Results

### 3.1. Quantitative Analysis

No missing values were identified in the data that was returned at baseline or post-intervention, however, several participants did not complete their final post-intervention questionnaire. To test whether there was any difference in program adherence between the groups, a new variable was created that coded participants as either having a full and complete dataset (across the two time points) or not. A chi-squared test of independence revealed a non-significant difference in program adherence between groups, X^2^ (2, *N* = 67) = 4.94, *p* = 0.09. Cross tabulation of participants with missing datasets across the three groups can be seen in Table 3.

Weekly participation rates for the active intervention groups were also tracked, as well as how often participants engaged with the task, as can be seen in Table 4.

For participants with complete baseline and post-interventions datasets, means and standard deviations for each variable of interest can be seen in Table 5.

In order to determine the impact of the interventions on owner-pet emotional closeness, mindfulness, and loneliness, a series of two-way mixed model ANOVAs were conducted. Studentized residuals were inspected for any values greater than ±3.29 and none found. Inspection of the data via normal and normal detrended Q-Q plots of studentised residuals revealed acceptable normal distribution of data. Levene’s test of equality of variances was used to determine equal variances between the categories of the groups at each time point. There was homogeneity of variances (*p* > 0.05) on all variables except the baseline 1-item loneliness measure and the baseline data for the measure of perceived emotional closeness. To account for this a stricter alpha level of 0.025 was applied to these analyses. Box’s test of equality of covariance matrices revealed there was homogeneity of covariances (*p* > 0.05) for all analyses. The main effects of group and time, as well as the group × time interaction effects can be found in the Table 6 below.

### 3.2. Qualitative Analyses

Qualitative analysis is presented in Table 7 and Table 8. Theme frequencies were calculated by summing together the number of participants who endorsed a theme. Percentages were calculated by dividing this number by the total number of participants who provided a response that week (minus those participants who provided a null response or a response that did not adequately address the question). Individual participants were only able to endorse a theme once but could endorse more than one theme. For the sake of presenting a summary, only those themes that were endorsed at a rate of 10% or greater for three or more weeks have been included in each Table. However, in addition to these, an individual week(s) may have had uniquely high endorsement of a theme not shown in this Table. For example, 25% of owners in the DI group endorsed a feeling of discomfort with the ‘talk time’ task: “This one felt a bit strange to me. I do talk to the dog but usually only for fun or commands”, with 19% commenting that there dog was restless during this task. This can be compared with 53% that said their dog was restless during the ‘picture time’ task. In the DAM group, the ‘dog’s fur’ mindfulness focus was perceived to be enjoyable for the dog for 11% in week 1 and 23% in week 3: “my dog was in heaven”. This highlights important differences between the individual tasks, and their relative effects on both owner and dog, in each group.

## 4. Discussion

This study aimed to evaluate the benefits of two novel owner–dog interactions that can be performed from home. Specifically, the study aimed to determine whether engaging in a DAM or DI six-week intervention could increase owner–dog attachment and owner mindfulness, and decrease owner loneliness, as compared to a control group. As expected, following the random allocation of participants to their group, there was no significant effect of group on any variable. Contrary to our hypothesis, there was no significant group × time interaction effect on any of the variables, however, there was a significant effect of time on loneliness, as measured by the UCLA-LS, whereby loneliness significant reduced over time. This lack of a group × time interaction effect is in contrast to positive dog interaction and DAM group effects reported previously [16,17]. Despite the lack of quantitative findings supporting group differences in the current study, qualitative insights revealed several encouraging owner-related themes such as ‘relaxed/calm’, ‘mindful/focussed/attentive/engaged, ‘fun/enjoyable/happy’ and ‘connected with dog’.

### 4.1. Dog Interaction Effects on Human–Dog Emotional Closeness

Owner–dog emotional closeness was not found to change over time in a quantitative way, despite qualitative reports of owner–dog connectedness during the activities (Table 7 and Table 8). Possible explanations for this include a lack of sensitivity of the MDORS emotional closeness subscale [20], with most participants across all groups scoring quite highly on this (refer to Table 5). This likely reflects that most people who self-selected into the study were dog lovers who already had a high level of emotional closeness with their dogs. As such, this lack of an increase may reflect ‘ceiling effects’ whereby the level of emotional closeness was already at such a high level that it was unable to be further increased with the intervention. In the case of the DI group, the lack of effect might be explained by the owner perceiving these weekly tasks to be normal activities they would regularly perform with their dog regardless of participating in the study. In conjunction with qualitative reports supporting this, this was also evidenced by the high mean number of times weekly tasks were completed, relative to the DAM group (refer to Table 4). However, there were also several instances of owners consciously having to put time aside to complete the tasks with their dogs (Table 8). It is interesting that this was not also endorsed in the DAM group, as they also must have had to put time aside to complete the task with the recording, and the task is likely to have been much more unusual than the tasks in the DI group. It is also interesting and somewhat concerning that some participants found it unusual to spend 7 undivided minutes of attention with their dogs, even with so many of them working from home during this time. While owners can enrich the lives of their dogs in other ways (e.g., providing them with toys, problem solving tasks, or time outside), findings from an Australian public perceptions study revealed that beliefs that companion dog happiness is dependent upon spending adequate time with their owners [30]. It is also interesting that 25% of owners in the DI group reported feelings of discomfort or awkwardness during the ‘talk time’ task (refer to Table 2 for task description). In line with Hockenhull and Furtado [2] we need to consider our pet keeping practices and how owners have a responsibility to enrich the lives of their pets. Doing something as simple as putting 7 min aside to attend to the pet seems like it might be a good place to start, and would be especially important for pets living in countries experiencing lockdowns, where going for walks is not permitted.

While both interventions clearly brought about a range of positive effects for the owners (refer to Table 7 and Table 8), a notable difference can be seen for the animal, with ‘Dog: fun/enjoyable/happy’ only being endorsed in the DI group. No other dog-related themes were endorsed sufficiently highly in either of the two groups for inclusion in the summary Tables. While we did not take any objective measures of the dog’s experience during the interventions, we can infer from the DI group reports that these activities were enjoyable for the dog. In particular, ‘hide and seek’, ‘follow the lead’ and ‘interactive play’. This theme was endorsed less in the later three activities, but this may be due to the fact that these were more calm activities where outward displays of happiness such as tail wagging, jumping, barking, etc., would be less common. However, it does not necessarily mean that the dog was not enjoying the one on one time with their owner, and indeed we see an increase of owner reported feelings of connectedness to their dogs during these activities, in particular ‘talk time’ (refer to Table 8). There was also uniquely high endorsement for ‘dog enjoyment’ in the ‘dog’s fur’ mindfulness task. This suggests that this specific mindfulness task might have benefits for both the owner and dog, as opposed to the other mindfulness tasks that appeared to be more beneficial for the owner than the dog, especially in week 6 when the dog was absent. Similarly, some of the interactions were perceived to be more enjoyable for the dogs than others, with dogs displaying signs of restlessness during ‘picture time’ and ‘talk time’. Although, it is difficult to know if the restless behaviour observed during talk time was in response to their owner’s feelings of discomfort with this task.

Interestingly in the DAM group, ‘connectedness to dog’ was more strongly endorsed when the object of focus was the dog’s fur or the dog’s breathing, and less so in the dogs’ heat sensation and body imagery objects of focus. This likely reflects the fact that the latter two mindfulness tasks were designed to be more challenging. Indeed, 64% of participants reported finding it difficult to focus during the imagery object of focus session and 0% reported feeling connected with their dog (Table 7). Unlike the earlier mindfulness tasks, week six practice did not require the presence of the participant’s dog, but rather, required the participant to recall their dog’s image using memory and/or imagination, which may have also impacted their ability to feel connected to their dog.

### 4.2. Dog Interaction Effects on Human Loneliness

The non-significant group × time interaction in relation to loneliness is in line with findings by Oliva and Johnston [12] who found that frequency of dog interactions were unrelated to loneliness scores. This might be explained by a recent study demonstrating that dog interactions are likely to impact positive affect but not negative affect [31]. The significant reduction in loneliness across time is likely to be explained by the timing of the study. For example, this study commenced 21 August 2020, however on 2 August the state of Victoria recorded 671 cases and seven deaths from COVID-19 resulting in the declaration of a state of disaster. Stage four restrictions were that day imposed on metropolitan-based Victorians, (which consisted of 79.1% of the total participants in the study, see Table 1) and stage 3 restrictions were imposed on rural Victorians 3 days later (10.4% of participants). As the numbers of COVID-19 cases dropped considerably during the latter half of September, the media were frequently communicating the possibility of the lockdown restrictions easing which may have given participants residing in Victoria a sense of hope and optimism. Furthermore, under Victoria’s roadmap of easing lockdown restrictions, individuals living alone (18% of the sample) were permitted to create a social bubble with one other person commencing September 14. Those in regional areas were also permitted to have outdoor public gatherings of up to 5 people. The stage four restrictions were lifted for Victorian metro participants on 28 September and the study concluded 2 October. As such, the timing of the study during the COVID-19 lockdown in Victoria, and the easing of these restrictions in the latter half of the study, may provide some understanding towards why a significant decrease in loneliness was recorded across the two time points. While this was only statistically significant when using the UCLA-LS, this is likely explained by the fact that we applied a stricter *p*-value to the 1-item direct measure, due to violations of homogeneity of variances.

### 4.3. Dog Interaction Effects on Human Mindfulness

The non-significant group × time interaction in relation to mindfulness is also in line with findings by Oliva and Johnston [12] that frequency of dog interactions was unrelated to mindfulness scores. However, this might be explained by the fact that instructions for the quantitative questions asked participants to reflect on their general state of mind during the preceding two weeks. In contrast, qualitative questions required participants to reflect specifically on the intervention tasks. As such, given the circumstances during which the study was conducted, we can potentially learn more about the impact of the interventions through the analysis of this intervention-specific qualitative data, than the quantitative data which might be subject to masking effects from the global pandemic situation. We can see from the qualitative reports that participants did feel a subjective feeling of attentiveness, focus, engagement, or mindfulness when participating in both tasks, and the presence of after-effects suggest that this feeling was able to be maintained for some time after the cessation of the task.

### 4.4. Strengths, Limitations, and Future Directions

The online delivery of the interventions via email was a notable strength during a time when people were either in lockdown or spending more time at home due to the COVID-19 pandemic. The inclusion of a control group was an important strength of the study, as time effects are likely to be highly sensitive to an event such as a global pandemic. However, the study would benefit from repetition during a ‘normal’ six-week time frame as the stress of the lockdown in Victoria might have masked more subtle positive effects of the intervention that we were unable to capture in a quantitative way. The social media recruitment strategy was also an effective campaign in securing participants in a short period of time, however, the pool of willing participants was biased due to the higher number of female responders, mostly from Monash University. Greater consideration for future studies to extend the research to other populations other than university employees/students would be recommended to further generalise results.

Apart from weeks 2 and 4 in the DAM group which repeated weeks 1 and 3, all other activities in both the DAM and DI groups varied. As such, it was difficult to monitor progression overtime, as some of the activities elicited unique experiences, e.g., increased restlessness in the dog in ‘talk time’ and ‘picture time’ and a majority preference for the ‘dog’s fur’ object of focus mindfulness task. Order effects when considering the activities may also be reflected in the qualitative responses to the tasks. Additionally, although some participants commented they enjoyed the ‘dog’s breathing’ mindfulness task, other participants provided comments suggesting they had tried to mimic their dogs breathing rhythms. This was not the intended purpose of the mindfulness practice, nor was it provided as an instruction (which stipulated that participants should be aware of their own breath, and follow their own rhythm). It is therefore understandable that participants found the task challenging and not beneficial, and this particular object of focus should perhaps be re-considered in future research studies. Future studies could also examine the acute and prolonged impact of individual mindfulness and interaction tasks in isolation.

The mindfulness tasks were designed to encourage participants to become more attentive and engaged when interacting with their pets, and we hoped that this would translate to interactions outside of the 7 min meditations (e.g., while stroking their dog’s fur when sitting and relaxing on the couch). However, according to the meditation training the first author has received, an object of focus should not change on a weekly basis, it should be maintained for at least 3 months. Further, an important figure of Siddha meditation, Muktananda [32], warned that meditating on an object other than the self or the divine may limit someone in what they can achieve from such a practice (e.g., “One who meditates on fish will always catch fish and eat fish and be peaceful and contented. Tell me, can you find God if you meditate on fish? Can you see the inner lights if you meditate on fish?”, p. 209). Therefore, while these activities may be a good way to encourage owners to interact with their pets more mindfully, they were not designed to help the participant achieve a state of spiritual enlightenment.

Finally, while we could presume that the weekly DI activities were enjoyable for the dogs, based on owner reports, future studies could extend these preliminary findings by evaluating benefits to dogs more objectively, e.g., with the use of behavioural evaluation and/or neurohormonal biomarkers (e.g., oxytocin, dopamine, prolactin, etc.), which have been associated with positive owner–dog interactions [7]. The same biomarkers could also be investigated in humans completing the tasks.

## 5. Conclusions

In conclusion, this study provides a demonstration of two easy to implement interventions: DAM and DI, that owners can complete with their dogs from the comfort of their homes. While there was no quantitative support for an increase in mindfulness, owner–dog emotional closeness or reduction in loneliness in the two active intervention groups, qualitative insights revealed that there were several common themes between the two interventions—including the owner feeling happiness/enjoyment and relaxation/calm while completing the task, as well as feeling a sense of engagement and/or focus while completing the task, and enhanced emotional/spiritual connection with their dogs. Positive after-effects were also experienced, which were often an extension of the positive sensations felt during the task.

## Figures and Tables

**Table 1:** Sociodemographic characteristics and participants’ mindfulness experience at baseline.

Baseline Characteristic	Dog Assisted Mindfulness (*n =* 21)	Dog Interaction (*n =* 23)	Control (*n =* 23)	Full Sample (*N =* 67)
**Gender**				
Male	2	4	2	8
Female	19	19	21	59
**State**				
Metropolitan Victoria (stage 4 restrictions)	19	16	18	53
Regional Victoria (stage 3 restrictions)	1	3	3	7
Queensland	1	1	1	3
New South Wales	0	1	1	2
Tasmania	0	1	0	1
**Living Conditions**				
I am unpartnered living with children	1	2	1	4
I live alone	3	5	5	13
I live with a partner	10	7	7	24
I live with family	5	8	9	22
I live with housemates	2	1	1	4
**Education**				
Postgraduate degree	12	11	5	28
Undergraduate degree	1	6	7	14
TAFE course or trade apprenticeship	4	4	9	17
Year 11/12	3	2	1	6
Year 10 or below	1	0	1	2
**Mindfulness experience**				
No experience	2	2	5	9
Brief experience (tried <10 times)	6	6	11	23
Inconsistent experience (tried 10 times or more but not consistently)	7	10	1	18
Frequent and consistent experience now or in the past for less than 6 months	4	1	3	8
Frequent and consistent experience now or in the past for more than 6 months	2	4	3	9

**Table 2:** Six week session activity description for Dog Interactions (DI) and Dog Assisted Mindfulness (DAM) groups.

Week	DI Group	DAM Group
1	**Hide and Seek:** Participants were instructed to hide somewhere in their house and call out to their dog to find them. Once found, they were instructed to praise them and repeat the task 3 times.	This week’s recording invited participants to engage with their **sense of touch** by placing their hand and fingertips on their dog, with the **object of focus being the dog’s fur**. If the dog walked away or became restless, participants were instructed to allow their dog to do as it pleases and continue with the recoding using their memory or imagination.
2	**Follow the Lead**: Participants were instructed to follow their dog’s lead doing anything they believed it wanted to do.	This week’s recording invited participants to engage with their **sense of sight**, with the **object of focus being the dog’s breathing**. Participants were initially instructed to keep their eyes softly open, and then close them towards the end of the session and continue their practice using visualisation techniques. If the dog walked away or became restless, participants were instructed to allow their dog to do as it pleases and continue with the recoding using their memory or imagination.
3	**Outside Interactive Play:** Participants were instructed to go outside with their dog and interact with them as they please. They were also told that the experience should be different to regular walking routine e.g., in the backyard or park.	Participants repeated the mindfulness activity as described in week 1, with the encouragement to delve deeper into this familiar practice.
4	**Affection Time:** Participants were simply instructed to provide their dog with 7 min affection time doing anything they please	Participants repeated the mindfulness activity as described in week 2, with the encouragement to delve deeper into this familiar practice.
5	**Picture Time:** With their phones on flight mode, participants were told to attempt to take the best selfie picture with their dog (inside or outside).	This week’s recording invited participants to engage with their **senses of touch and inner sight**, by closing their eyes and placing their hand on their dog with the **object of focus being the dog’s heat sensation**. They were instructed to visualise the heat as a bright light, and encouraged to feel it with their physical touch. If the dog walked away or became restless, participants were instructed to allow their dog to do as it pleases and continue with the recoding using their memory or imagination.
6	**Talk Time:** Participants were instructed to sit comfortably facing their dog and talk or read to them.	Participants were invited them to engage with their **sense of inner touch**, this time with their dog outside the room, with the **object of focus being the dog’s body imagery**. They were instructed to close their eyes and tap into this inner sense using their memory/imagination while mentally tracing an outline of their dog in their mind’s eye, using/imagining their hand, and connecting to any feelings of warmth if they were present.

Note: all sessions were approximately 7 min in length.

**Table 3:** Complete datasets group crosstabulation.

Complete Datasets	Dog Assisted Mindfulness		Dog Interaction Group		Control	
	*n*	%	*n*	%	*n*	%
Yes	12	57.1%	19	82.6%	19	82.6%
Standardised Residuals	−0.9		0.4		0.4	
No	9	42.9%	4	17.4%	4	17.4%
Standardised Residuals	1.6		−0.8		−0.8	
Total	21	100%	23	100%	23	100%

N = 67.

**Table 4:** Mean number of times weekly tasks were completed per participant in the active intervention groups.

	Week 1		Week 2		Week 3		Week 4		Week 5		Week 6	
	*M (SD)*	*n*	*M (SD)*	*n*	*M (SD)*	*n*	*M (SD)*	*n*	*M (SD)*	*n*	*M (SD)*	*n*
Interaction	4.4 (5.0)	21	3.8 (4.3)	19	4.5 (4.6)	20	8.6 (9.2)	18	4.3 (4.5)	17	5.1 (6.1)	19
Mindfulness with recording	2.0 (1.5)	22	1.6 (1.3)	19	2.3 (1.5)	13	1.6 (1.5)	15	1.8 (1.5)	14	1.6 (1.6)	15
Mindfulness without recording	1.7 (1.8)	22	1.9 (1.9)	19	2.1 (2.5)	13	1.3 (1.3)	15	1.1 (1.5)	14	1.1 (1.6)	15

**Table 5:** Descriptive statistics for participants with complete data.

		Mean at Baseline	*SD*	Mean Post-Intervention (week 7)	*SD*	*N*
Emotional Closeness	Control	4.05	0.46	4.10	0.43	19
	Interaction	3.98	0.44	4.09	0.39	19
	Mindfulness	3.92	0.73	3.93	0.62	12
Mindfulness	Control	36.32	6.33	36.32	6.39	19
	Interaction	36.79	8.01	37.74	8.53	19
	Mindfulness	34.75	7.10	35.25	6.51	12
UCLA-LS	Control	6.89	2.85	5.58	2.32	19
	Interaction	6.21	2.02	6.00	2.49	19
	Mindfulness	7.75	2.30	6.00	3.67	12
Loneliness 1-item	Control	2.11	0.99	1.79	0.86	19
	Interaction	1.74	0.73	1.79	0.95	19
	Mindfulness	2.42	1.31	2.00	1.21	12

Note: The emotional closeness sub-scale ranges from range from 1–5 with higher scores indicating higher emotional closeness, mindfulness scores range between 14–56 with higher scores indicating higher mindfulness, UCLA-LS scores range between 3–12 with higher scores indicating higher loneliness, the 1-item measure of loneliness ranges from 1–4, with higher scores indicating higher loneliness. *N =* 50.

**Table 6:** Mixed methods ANOVA results for group, time, and group × time interaction effects.

		*F*	*p*	Partial Eta
Emotional Closeness	Group	0.41	0.67	0.017
	Time	0.77	0.38	0.016
	Group × Time	0.24	0.78	0.010
Mindfulness	Group	0.40	0.67	0.017
	Time	0.55	0.46	0.011
	Group × Time	0.21	0.81	0.009
UCLA-LS	Group	0.42	0.66	0.018
	Time	12.01	0.001 *	0.204
	Group × Time	2.17	0.13	0.085
Loneliness 1-item	Group	1.16	0.32	0.047
	Time	4.50	0.04	0.088
	Group × Time	0.80	0.46	0.033

*N =* 50, * *p* < 05.

**Table 7:** Most frequently endorsed themes relating to the dog assisted mindfulness groups weekly task experiences.

Theme	Definition	Example	Week 1 Task: Dogs Fur (*n =* 20)	Week 2 Task: Dogs Breathing (*n =* 18)	Week 3 Task: Dogs Fur (*n =* 13)	Week 4 Task: Dogs Breathing (*n =* 14)	Week 5 Task: Heat Sensation (*n =* 13)	Week 6 Task: Body Imagery (*n =* 11)
Owner: Relaxed/Calm	Task induced feelings of relaxation or calm	Overall, very relaxing. Lots of head tingles!	13 (65%)	7 (39%)	7 (54%)	6 (43%)	6 (46%)	2 (18%)
Owner: Connected with Dog	Owner’s spiritual/emotional connection with dog increased	Felt connected, like it was just the 2 of us, no outside interference	8 (40%)	7 (39%)	2 (15%)	2 (14%)	1 (8%)	0 (0%)
Owner: After Affects	Effects of the task continued to be felt by the owner after the cessation of the activity	Immediate feelings of love and connection for duration of the exercise and for a few hours or so later	6 (30%)	2 (11%)	2 (15%)	2 (14%)	1 (8%)	1 (9%)
Owner: Difficulty to Focus ^†^	Owner experienced mental distractions/interuptions whilst participating in activty	I find it difficult to focus on my dog’s breathing and not be distracted. I found myself getting a little frustrated	4 (20%)	2 (11%)	3 (23%)	1 (7%)	2 (15%)	7 (64%)
Owner: Mindful/Focused/Attentive/Engaged.	Owner achieved a focused/attentive/mindful or engaged state of mind whilst participating in the task	Watching her breathing helped me focus on one thing	2 (10%)	1 (6%)	4 (31%)	4 (29%)	5 (38%)	3 (27%)
Owner: Anticipation ^†^	Owner thought about/anticipated the task prior to completing it	Looked forward to doing it as the week 1 exercise had been so pleasant	2 (10%)	2 (11%)	3 (23%)	1 (7%)	0 (0%)	0 (0%)
Owner: Fun/Enjoyable/Happy	Owner felt the task was fun/enjoyable or felt happy completing it	It’s nice to involve them in this exercise of wellbeing	1 (5%)	2 (11%)	3 (23%)	1 (7%)	5 (38%)	0 (0%)

^†^ Theme unique to group. Frequencies are calculated as the number of people who endorsed a theme that week. Each participant could endorse more than one theme but could endorse the same theme only once per week.

**Table 8:** Most frequently endorsed themes relating to the dog interaction groups weekly task experiences.

Theme	Definition	Example	Week 1 Task Hide and Seek (*n =* 21)	Week 2 Task Follow the Lead (*n =* 18)	Week 3 Task Interactive Play (*n =* 20)	Week 4 Task Affection Time (*n =* 17)	Week 5 Task Picture Time (*n =* 17)	Week 6 Task Talk Time (*n =* 16)
Owner: Fun/Enjoyable/Happy	Owner felt the task was fun/enjoyable or felt happy completing it	Another hilarious activity that made me literally laugh out loud	17 (81%)	12 (67%)	15 (75%)	6 (35%)	7 (41%)	5 (31%)
Dog: Fun/Enjoyable/Happy ^†^	Owners percieved their dog enjoyed the participating in the task with them	Her tail was wagging and she was barking with excitement	12 (57%)	6 (33%)	8 (40%)	3 (18%)	1 (6%)	1 (6%)
Owner: Connected with Dog	Owner’s spiritual/emotional connection with dog increased	Connected. I can see doing this often may strengthen your relationship with your pet	4 (19%)	0 (0%)	4 (20%)	7 (41%)	2 (12%)	10 (63%)
Owner: After Affects	Effects of the task continued to be felt by the owner after the cessation of the activity	My mood uplifted after playing with him	4 (19%)	2 (11%)	3 (15%)	3 (18%)	1 (6%)	1 (6%)
Owner: Rewarding ^†^	Task was rewarding to owner in unexpected ways	Made me feel about 20 years younger running around	2 (10%)	1 (6%)	5 (25%)	0 (0%)	5 (29%)	2 (13%)
Owner: Mindful/Focused/Attentive/Engaged	Owner achieved a focused/attentive/mindful or engaged state of mind whilst participating in the task	I do generally give my dog plenty of affection time already but it was good to be more focussed on it rather than mindlessly doing so	1 (5%)	4 (22%)	3 (15%)	2 (12%)	0 (0%)	0 (0%)
Owner Putting Time Aside ^†^	Owner consciously had to plan/put time aside to complete the task	I had to make time to do it…it was good because it moved me away from my desk	0 (0%)	4 (22%)	1 (5%)	2 (12%)	0 (0%)	2 (13%)
Owner: Relaxed/Calm	Task induced feelings of relaxation or calm	This was a lovely experience. It felt calming and gentle	0 (0%)	0 (0%)	3 (15%)	6 (35%)	2 (12%)	0 (0%)

^†^ Theme unique to group. Frequencies are calculated as the number of people who endorsed a theme that week. Each participant could endorse more than one theme but could endorse the same theme only once per week.

## Data Availability

The data presented in this study are available on request from the corresponding author.
